# Zebrafish as a Model System for Brugada Syndrome

**DOI:** 10.31083/j.rcm2509313

**Published:** 2024-09-05

**Authors:** Leonie Verkerk, Arie O. Verkerk, Ronald Wilders

**Affiliations:** ^1^Department of Medical Biology, Amsterdam Cardiovascular Sciences, Amsterdam UMC, University of Amsterdam, 1105 AZ Amsterdam, The Netherlands; ^2^Department of Experimental Cardiology, Heart Center, Amsterdam Cardiovascular Sciences, Amsterdam UMC, University of Amsterdam, 1105 AZ Amsterdam, The Netherlands

**Keywords:** Brugada syndrome, zebrafish, *Danio rerio*, human, heart, ventricle, cardiomyocytes, electrophysiology, action potential, sodium current

## Abstract

Brugada syndrome (BrS) is an inheritable cardiac arrhythmogenic disease, associated with an increased risk of sudden cardiac death. It is most common in males around the age of 40 and the prevalence is higher in Asia than in Europe and the United States. The pathophysiology underlying BrS is not completely understood, but several hypotheses have been proposed. So far, the best effective treatment is the implantation of an implantable cardioverter-defibrillator (ICD), but device-related complications are not uncommon. Therefore, there is an urgent need to improve diagnosis and risk stratification and to find new treatment options. To this end, research should further elucidate the genetic basis and pathophysiological mechanisms of BrS. Several experimental models are being used to gain insight into these aspects. The zebrafish (*Danio rerio*) is a widely used animal model for the study of cardiac arrhythmias, as its cardiac electrophysiology shows interesting similarities to humans. However, zebrafish have only been used in a limited number of studies on BrS, and the potential role of zebrafish in studying the mechanisms of BrS has not been reviewed. Therefore, the present review aims to evaluate zebrafish as an animal model for BrS. We conclude that zebrafish can be considered as a valuable experimental model for BrS research, not only for gene editing technologies, but also for screening potential BrS drugs.

## 1. Introduction

Brugada syndrome (BrS) is a rare inherited cardiac disorder with an estimated worldwide prevalence of 0.5 per 1,000 individuals [1]. The age of onset is highly variable, but symptoms usually appear around the age of 40 [2, 3, 4, 5, 6, 7]. BrS was named after the two cardiologist brothers Pedro and Josep Brugada who, in 1992, characterized a group of eight patients with an elevated ST segment in precordial leads V_1_–V_3_ of their electrocardiogram (ECG) during sinus rhythm, together with right bundle branch block (RBBB) and a normal QT interval, in the absence of electrolyte disturbances, ischemia, or structural cardiac abnormalities [8]. In a follow-up study published in 1998, the three cardiologist brothers Josep, Ramon, and Pedro Brugada analyzed data from 63 patients with this ECG pattern identified in 33 centers around the world [9]. Interestingly, BrS was found mainly in male patients (56 male, 7 female). Later, it became clear that the ECG abnormalities typical for BrS were already observed in several other studies before 1992, including the 1953 study by Osher and Wolff [10], as reviewed by Naccarelli and Antzelevitch [11]. However, the syndrome was not renamed.

Patients with BrS have an increased risk of syncope and sudden cardiac death, which are often the first signs of BrS [4, 5, 6, 7, 12], underscoring the importance of early diagnosis and proper risk stratification [13]. However, more than 60% of BrS patients are asymptomatic at the time of diagnosis [14, 15, 16], highlighting the challenge of identifying BrS at an early stage. It should be noted that this percentage has been challenged by Viskin *et al*. [17] and may actually be substantially lower. An important strategy that has been shown to be effective in preventing sudden cardiac death is the use of an implantable cardioverter-defibrillator (ICD), an electronic device that can detect and correct irregular heartbeats [2, 4, 5, 6]. Pharmacological treatment with class 1A antiarrhythmic drugs, particularly quinidine, has also been shown to be successful [18, 19, 20], as reviewed by Brodie *et al*. [21]. In the 2022 ESC Guidelines, ICD implantation is recommended for high-risk patients, while quinidine should be considered for high-risk patients who have an ICD contraindication, are declining, or have recurrent ICD shocks [22]. However, both ICD implantation and the use of quinidine are associated with specific problems [22]. These aspects highlight the need for improved identification and risk stratification of BrS, as well as the urge to develop new preventive strategies against sudden cardiac death. Since the 1992 paper of the Brugada brothers [8], subsequent research has contributed to the understanding of the causes and mechanisms of BrS. *SCN5A * (human sodium voltage-gated channel alpha subunit 5 gene) was found to be the major BrS-associated gene. This gene encodes the pore-forming α*-*subunit of the cardiac fast sodium channel and it is mutated in 20–30% of BrS patients [2, 22], which can eventually lead to sudden cardiac arrest due to ventricular arrhythmias [4, 5, 6]. In the vast majority of cases, these arrhythmias are polymorphic ventricular tachycardia (VT) or ventricular fibrillation (VF), but monomorphic VT has also been observed (see Rodríguez-Mañero *et al*. [23] and primary references cited therein). Despite this finding, the cause of BrS is still unknown in most diagnosed patients [24]. This example shows that research on BrS has progressed since 1992, but that information is still limited.

In order to improve the quality of life of patients and prevent life-threatening situations, it is important to continue to gain knowledge about BrS. Model systems exemplify how the pathophysiological mechanisms of BrS can be studied. These systems include expression systems for (mutant) ion channels, such as human embryonic kidney (HEK) cells, but also murine, canine, and porcine animal models, as well as human induced pluripotent stem cell-derived cardiomyocytes (hiPSC-CMs). Each of these models has its own benefits and limitations [24, 25, 26]. Another popular animal model system used in studies on cardiac arrhythmias is the zebrafish (*Danio rerio*) [27]. This fish is known to share characteristics with humans in terms of cardiac electrophysiology, thus providing unique opportunities for the study of arrhythmogenic diseases [28, 29, 30]. In addition, zebrafish are known to have at least one obvious ortholog for 71% of the human genes [29]. Therefore, zebrafish could provide new insights into BrS in particular. However, a PubMed and Google Scholar literature search for ‘Brugada’ and ‘zebrafish’ in combination only revealed the studies of Zhou *et al*. [31], Juang *et al*. [32], Barc *et al*. [33], and Chiang* et al*. [34], from 2016, 2020, 2022, and 2024, respectively, in which zebrafish have actually been used as a model system for BrS thus far. This makes it understandable that the potential application of zebrafish in research on BrS has not yet been discussed in reviews of BrS model systems [24, 26]. Therefore, in the present paper, we ourselves review zebrafish as a model system for BrS research.

## 2. Electrophysiology of the Brugada Syndrome

### 2.1 Characteristics of the Brugada Syndrome

#### 2.1.1 Clinical Characteristics

**Fig. 1.**
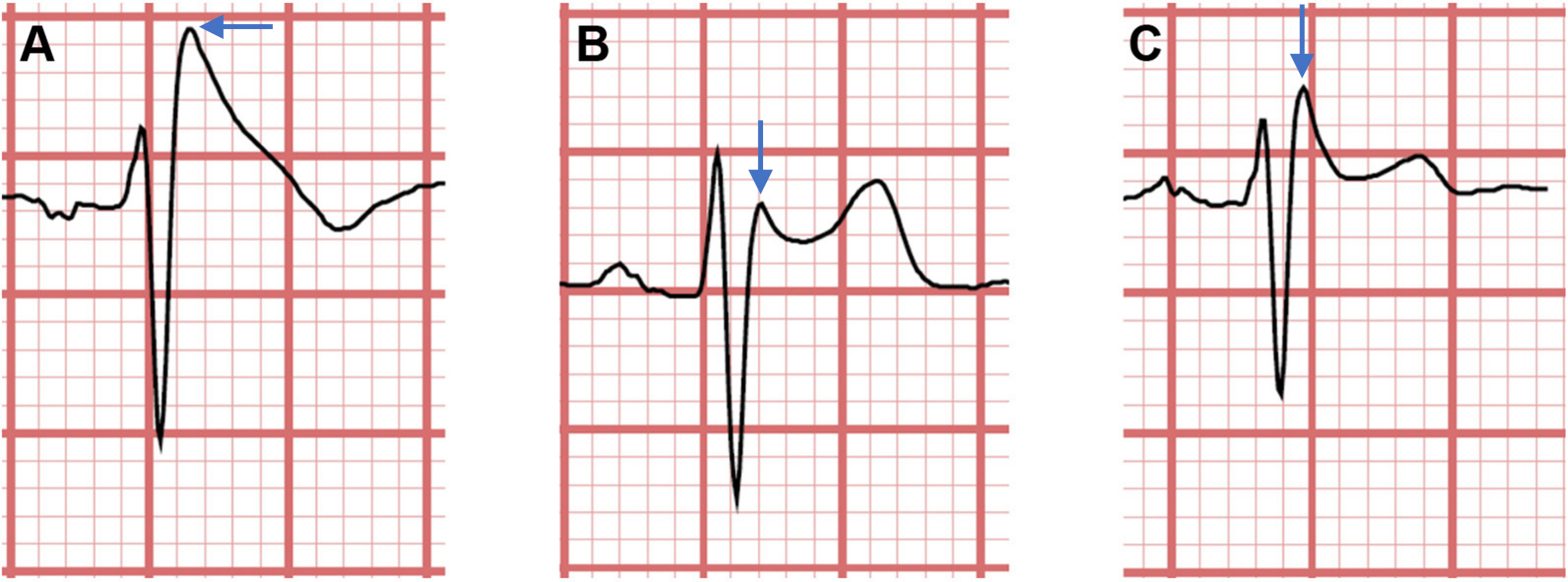
**Brugada syndrome electrocardiogram (ECG) types 1, 2, and 3**. (A) ECG type 1, featuring a coved ST segment with a T-wave inversion. Only type 1 is considered diagnostic. (B) ECG type 2, featuring a saddleback ST-configuration, of which the terminal portion (latter half of the ST segment) is elevated ≥1 mm. (C) ECG type 3, featuring a saddleback ST-configuration, of which the terminal portion is elevated <1 mm. Arrows indicate the J point, *i.e.*, the junction where the QRS complex merges with the ST segment, in each of the registrations. All ECG types have ≥2 mm elevation of the J point from baseline in the chest leads V_1_, V_2_, or V_3_. Reproduced from Krahn *et al*. [4] with permission.

BrS was initially characterized by a peculiar ECG pattern, featuring RBBB and elevation of the ST segment in leads V_1_ through V_3_ of the ECG (Fig. 1, Ref. [4]) [8]. An RBBB refers to a conduction block in the cells of the His-Purkinje fibers that excite the right ventricle, affecting the sequence of depolarization, usually observed as a broadened QRS complex on the ECG (>120 ms, for a complete RBBB). However, most BrS patients show a pseudo-RBBB, and thus only show the corresponding, typical ECG pattern [35]. An ST-elevation refers to the ST segment of the ECG being abnormally high above baseline, and may be associated with BrS when ischemia, electrolyte imbalance, inflammatory disease, and nervous system disorders are absent. As the underlying mechanisms of BrS remain disputable, the more appropriate term idiopathic ST-elevation could be used [36, 37]. BrS is also characterized by an increased risk of syncope, VF, and therefore sudden cardiac death [4, 6].

#### 2.1.2 Diagnosis

Initially, three types of ECG patterns were associated with BrS, but criteria to diagnose BrS have evolved over time [5]. As a consequence, type 3 is not in common use today and has been combined with type 2 in recent studies (*e.g.*, [4, 38, 39]). Type 1 is considered to be diagnostic [5, 22]. This type is characterized by a coved ST-elevation with inversion of the T wave, whereas types 2 and 3 include a saddleback ST elevation with varying degrees of elevation (Fig. 1) [2, 4, 40]. More than 60% of BrS patients are asymptomatic when diagnosed [14, 15, 16] (keeping in mind that this high percentage has been challenged by Viskin *et al*. [17] as set out in the Introduction), but these individuals are evaluated through routine screening, in which type 2 and 3 ECG patterns are indicative for BrS, or these patients are suspected of having BrS based on their clinical or family history. Further evaluation is done by provocative testing using Class 1 agents, *i.e.*, sodium channel blockers, to induce arrhythmogenic symptoms [4]. Class 1A and 1C, but not 1B, antiarrhythmic drugs have been found to reinforce the pathophysiological changes of the ST segment in BrS patients [41], but only infrequently (2–4%) in healthy subjects [22].

Currently, ajmaline, procainamide, flecainide, and pilsicainide are used for provocation tests for BrS, depending on the national availability of these drugs. These drugs are known to block the cardiac Na_V_1.5 voltage-gated sodium channel (Na_V_1.5 channel, named after its pore-forming α-subunit Na_V_1.5) and thereby inhibit the cardiac fast sodium current (I_Na_). However, the sensitivity and accuracy of these drugs differ [4, 42]. Furthermore, the presence of a drug-induced type 1 ECG *per se* has been questioned as sufficient for the diagnosis of BrS [42, 43]. Therefore, the more recent Shanghai Score System includes additional criteria that take into account the patient’s ECG pattern, clinical history, family history, and genetic test results [44]. Individuals receive a score indicating whether BrS is probable or definite (≥3.5 points), possible (2–3 points), or whether a patient is non-diagnostic (<2 points). A retrospective analysis validated this score system, finding that increasing scores were associated with increasing frequencies of VT and/or VF events [44].

#### 2.1.3 Epidemiology

Because the diagnosis of BrS is quite challenging, estimating the prevalence of this syndrome has also been difficult [2, 7]. Nevertheless, the prevalence of BrS has been shown to be higher in Asia than in Europe and the United States [1, 45]. The prevalence of BrS in the pediatric population is remarkably low (0.005%) compared to the adult population (estimated worldwide prevalence of 0.05% [1]), and no sex predominance has been found among diagnosed children [4, 46]. However, after adolescence, 80% to 90% of diagnosed patients are male [1]. This, together with the normalization of ST-elevation after surgical castration in two BrS patients with prostate cancer, suggests a possible role of testosterone in the pathophysiology of BrS [47, 48]. Regarding the average age at which the first arrhythmic event occurs, Alings and Wilde [49] observed a peak around the fourth decade.

### 2.2 Pathophysiology of the Brugada Syndrome

#### 2.2.1 Brugada Syndrome as a Channelopathy

Dysfunction of ion channels can disrupt the function of multiple tissues, resulting in diseases commonly referred to as ion channelopathies [50]. BrS has been described as an ion channelopathy as well, and has been divided into several types depending on which gene is altered. However, recent studies have questioned the underlying cause of BrS, as causative relationships between genetic alterations and a BrS phenotype have not been established in many cases [51, 52]. In 2016, BrS had already been classified into more than 20 types, the most prevalent of which are due to loss-of-function mutations in genes encoding proteins related to the Na_V_1.5 sodium channel and the Ca_V_1.2 calcium channel [53]. BrS has been described as an autosomal dominant inheritable disorder [54], meaning that the relevant mutated genes are located on non-sex chromosomes, and that receiving only one copy of the mutated gene can cause the phenotype of the disease. To date, 23 genes have been described to be associated with the BrS phenotype [7]. In addition, it has been shown that the electrophysiological abnormalities of the right ventricular myocardium are mainly determined by the genetic background of the patient, as the genetics of BrS are associated with the severity of the phenotype [55].

Genes associated with BrS are not restricted to the aforementioned *SCN5A*, but also include, for example, *CACNA1C *(human calcium voltage-gated channel subunit alpha1 C gene) and *CACNB2b* (human voltage-gated calcium channel auxiliary subunit beta 2b gene), which encode the β1c and β2b β-subunits of the cardiac Ca_V_1.2 calcium channel, respectively. In addition, gain-of-function mutations in genes encoding proteins constituting the channels that carry the transient outward potassium current (I_to_) have also been shown to be associated with BrS [53]. In total, more than 300 different mutations have been shown to be associated with BrS [56]. However, with the exception of the aforementioned mutations in *SCN5A* observed in 20–30% of the BrS cases, all of these mutations are expected to be responsible for only 5% of the BrS cases, and >65% of BrS cases have not been shown to originate from genetic abnormalities at all [2]. As emphasized above, no clear genotype-phenotype correlation has yet been established for these non-*SCN5A* mutations and their causative role in BrS is therefore highly debated [51, 52].

As noted above, *SCN5A* is the most commonly mutated gene in BrS patients, with loss-of-function mutations present in 20–30% of BrS cases [2, 4, 7, 22, 53, 54]. Moreover, *SCN5A* is the only gene for which a genotype-phenotype correlation has been demonstrated [7, 51, 52]. A wide variety of mutations in this gene are known to significantly affect the characteristics of the cardiac fast sodium channel, resulting in a less effective I_Na_ due to changes in its current density and/or kinetics. The changes in kinetics include a delayed activation, an accelerated inactivation, or a combination of both [57].

#### 2.2.2 Hypotheses on Pathophysiology 

**Fig. 2.**
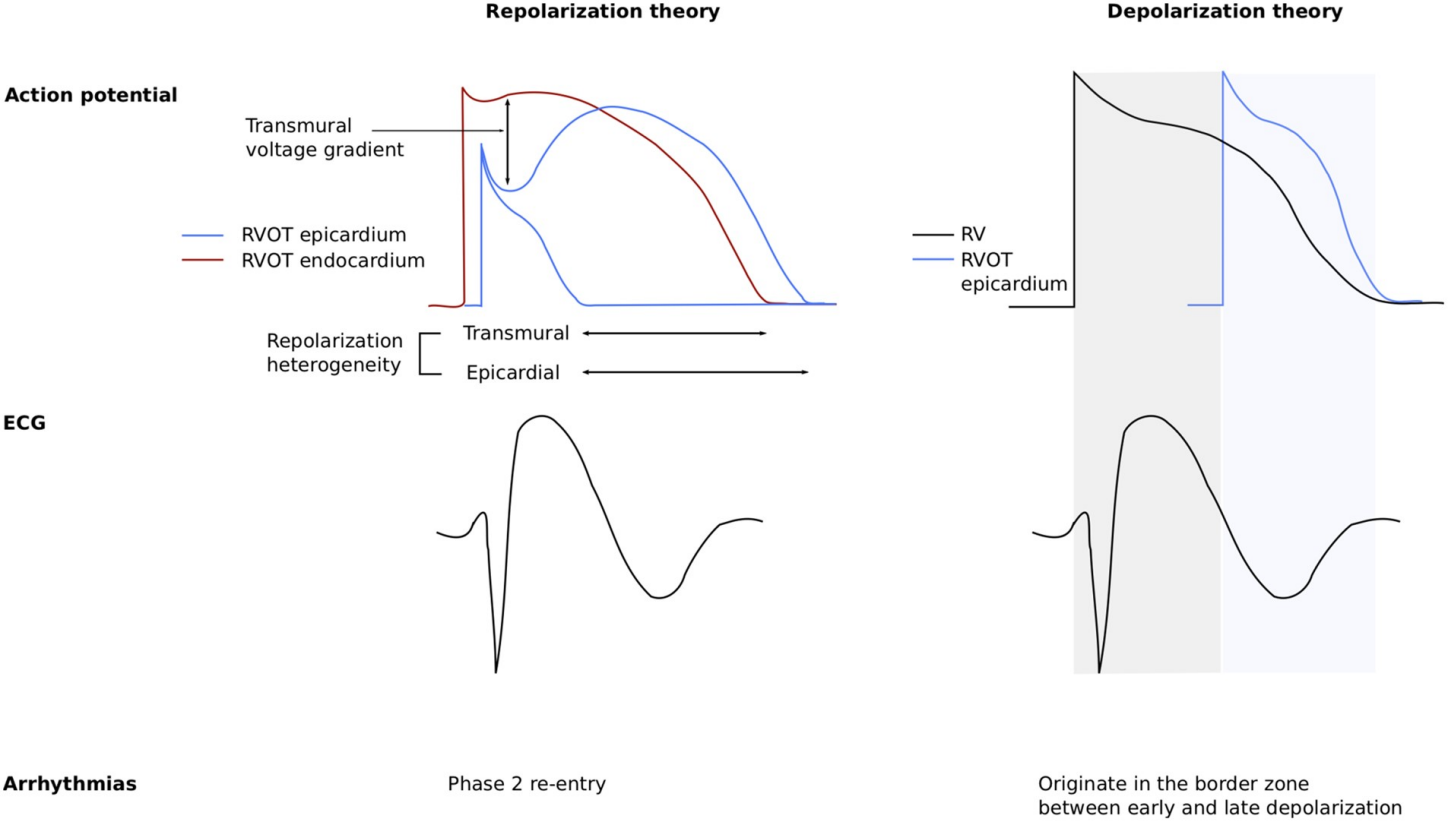
**The repolarization and depolarization theories of BrS pathophysiology**. The repolarization theory (left) states that an impaired sodium current (I_Na_) induces an enhanced role of the transient outward potassium current (I_to_) due to the smaller opposing inward currents (an impaired I_Na_ and a less strongly activated L-type calcium current), especially in the epicardium of the right ventricular outflow tract (RVOT) as compared to the RVOT endocardium (blue and red traces, respectively), underlying a loss of dome of the action potential of the RVOT epicardial cells (top left). This results in a transmural voltage gradient between the epicardium and endocardium of the RVOT, which explains the typical BrS ECG (bottom left) and facilitates arrhythmias through phase 2 re-entry. The depolarization theory (right) is based on delayed electrical conduction in the RVOT, due to a reduced I_Na_ and structural alterations of mainly the epicardial layers in the RVOT, causing mismatched action potentials in the right ventricle (RV) and the RVOT (top right), explaining the typical BrS ECG (bottom right) and facilitating arrhythmias in the border zone between early and late depolarization. Reproduced with permission from Behr *et al*. [54]. BrS, Brugada syndrome; ECG, electrocardiogram.

The mechanism by which a reduced I_Na_ function leads to the clinical features of BrS is controversial and involves several hypotheses [5, 57]. The major two theories explain the pathophysiology of BrS by either a depolarization disorder or a repolarization disorder (Fig. 2, Ref. [54]). The depolarization hypothesis proposes that a delayed electrical conduction in the right ventricular outflow tract (RVOT), as a result of the impaired I_Na_ and structural alterations of mainly the epicardial layers, is the underlying mechanism of BrS, supported by the presence of late action potentials in BrS patients. Thus, arrhythmias are thought to result from mismatched action potentials originating at the boundary between early and late depolarization (Fig. 2, right). This is in contrast to the repolarization theory, which proposes that an epicardial-endocardial transmural voltage gradient underlies the typical BrS ECG. This gradient is caused by the reduced I_Na_, resulting in a relatively increased I_to_. Because I_to_ is more pronounced in the epicardium of the right ventricle, as compared to the endocardium, an accentuated phase 1 repolarization can only be observed in the epicardium of the RVOT. As this leads to heterogeneity in the local repolarization of the right ventricular cardiomyocytes, there is an increased risk of phase 2 re-entry, and thus for VT and/or VF. Although evidence has been found for both hypotheses separately, it cannot be excluded that a combination of both mechanisms underlies the pathophysiology of BrS [4, 7, 54]. Moreover, a combination of both depolarization and repolarization abnormalities has been demonstrated in BrS patients [58, 59]. In fact, Yokokawa *et al*. [60] examined the spatial distribution of depolarization and repolarization disorders in BrS patients and concluded that depolarization disorders occur homogeneously throughout the ventricular wall, and that repolarization disorders only occur in the region of the RVOT.

#### 2.2.3 A more Complex Inheritance and a Contribution of Environmental Factors

Since no causative genetic mutation can be found in >65% of BrS cases, BrS is expected to follow a more complex inheritance pattern than initially thought. There is increasing evidence that BrS is an oligogenic or polygenic disease rather than a monogenic disease, meaning that multiple genetic factors underlie the BrS phenotype [24, 37, 53]. For example, a genome-wide association study by Bezzina *et al*. [61] revealed three loci, near *HEY2* and at *SCN5A-SCN10A*, that had an unexpectedly large cumulative effect on BrS susceptibility. In addition, in a family with two missense mutations in *SCN5A *(P336L and I1660V), a BrS phenotype was observed only in the proband who carried both mutations, but not in four family members who carried only one of these mutations [62].

BrS is known to show incomplete penetrance, meaning that not every individual with a BrS-associated genotype also shows the BrS phenotype. This indicates the impact of environmental factors on the genetic predisposition for BrS [51]. Although BrS defines ST-segment elevation in patients with structurally normal hearts, it does not signify that subclinical structural changes cannot be observed in BrS patients. As a matter of fact, BrS-related right ventricular structural changes had already been noted in the 1980s [63, 64, 65] and subclinical structural changes have been observed in a significant proportion of patients with BrS [66, 67]. So, structural abnormalities, especially in the RVOT, may further contribute to the development of the BrS phenotype and therefore contribute to ventricular arrhythmias [7]. Besides structural abnormalities, other factors are known to induce Brugada phenocopy, a term to be distinguished from actual BrS. Brugada phenocopy refers to the typical BrS ECG pattern, but which may be reversible, and which often does not require invasive treatment. Conditions known to induce Brugada phenocopy include right ventricular ischemia, pericarditis, hypo- and hyperkalemia, and acute pulmonary embolism [45, 68].

### 2.3 Risk Stratification and Clinical Management of the Brugada Syndrome

#### 2.3.1 Risk Stratification

As already noted when it was first described, BrS is associated with an increased risk of sudden cardiac death [4, 5, 6, 7, 8, 12]. However, not all patients are at the same risk of life-threatening events. Risk stratification provides a better understanding of which patients are at higher risk of serious arrhythmic events and thus require treatment. Asymptomatic patients are known to have the lowest risk of cardiac events. A risk of cardiac events of 0.5% and 0.4% per year was observed by Probst *et al*. [16] and Delise *et al*. [69], respectively. Their groups of asymptomatic patients included both patients with a spontaneous type 1 ECG and patients with a drug-induced type 1 ECG. In more recent studies, *e.g.*, those by Probst *et al*. [70] and Gaita *et al*. [71], separate risks were calculated for these two groups of patients. With values of 0.26% *vs*. 0.61% per year [70] and 0.03% *vs*. 0.4% per year [71], the risk for asymptomatic patients with a drug-induced type 1 ECG is lower than for asymptomatic patients with a spontaneous type 1 ECG. The overall risk for these two patient groups in the studies by Probst *et al*. [70] and Gaita *et al*. [71] was 0.36 and 0.2%, respectively. In symptomatic patients, clinical variables that have a large impact on risk stratification, are resuscitated cardiac arrest, cardiogenic syncope, and the presence of a spontaneous ECG type 1 pattern [4, 24, 70, 71]. Family history and *SCN5A* mutations have been shown to have no predictive value for VT and VF [16]. Furthermore, despite the male predominance seen in the prevalence of BrS, Probst *et al*. [16] revealed that sex does not have a significant impact on the prognosis of cardiac events. However, Sieira *et al*. [72] demonstrated a less severe clinical presentation and a more favorable prognosis for women compared to men. Together with the disputable role of age in risk stratification [73], it is clear that the potential impact of certain factors requires clarification [3, 4, 12].

#### 2.3.2 Clinical Management

Risk stratification is important to provide the most accurate management for a specific BrS patient. Advising conservative measures is the first step in preventing cardiac events and is often sufficient for asymptomatic patients diagnosed after provocation tests. Such conservative measures include avoidance of arrhythmogenic drugs, encompassing alcohol and over-the-counter medications, and aggressive treatment of fever [4, 12]. The recommended and proven effective treatment for high-risk patients is the implantation of an ICD [2, 3, 4, 5, 6, 7, 12, 22]. However, implantation of an ICD is not without consequences; device-related complications have a prevalence as high as 8.9% per year [74], not to mention the psychological and social consequences of carrying an ICD [4]. Epicardial ablation of the arrhythmic substrate appears to be a promising alternative treatment [75, 76, 77, 78, 79], as reviewed by Hoeksema *et al*. [5], Nademanee *et al*. [80], and Chokesuwattanaskul and Nademanee [81, 82].

The pharmacological treatment of BrS is based on the application of drugs that are supposed to restore the imbalance of cardiac ionic currents in BrS patients, of which quinidine is an example. Although quinidine has been shown to reduce several inward and outward currents, including the L-type calcium current (I_CaL_) [83] and the rapid and slow delayed rectifier potassium currents (I_Kr_ and I_Ks_, respectively) [84], the main effect is achieved through the inhibition of I_to_[85]. Its overall effect is to prolong the refractory period of the cardiomyocytes, and therefore quinidine has antiarrhythmic characteristics [4]. However, quinidine has not yet been included in first-line therapy because side effects are a major problem with quinidine administration. Examples of adverse reactions are diarrhea, potentially leading to ventricular arrhythmias due to electrolyte imbalances, and torsade de pointes, a ventricular arrhythmia that can lead to sudden cardiac death [86]. In addition, the lack of availability of quinidine creates a medical burden on a global scale [87]. Pharmacological alternatives, such as cilostazol [88] or orciprenaline [89], are being investigated, but evidence on the efficacy and safety of these drugs remains insufficient [86]. Overall, although important progress has been made, the clinical management of BrS remains challenging [90, 91, 92, 93, 94, 95, 96].

### 2.4 The Brugada Syndrome in Summary

In summary, since BrS was described by Pedro and Josep Brugada in 1992 [2], significant progress has been made in the research of this disease. Improvements on diagnostics have been made and more insight has been gained into the epidemiology of BrS. Research has been conducted on the genetics of BrS and researchers have gained insight into the pathophysiological mechanisms underlying BrS. In addition, risk stratification ensures that informed decisions can be made about how to manage BrS specifically in each patient. Unfortunately, however, there are still gaps in knowledge about all these aspects. What is the most reliable way to diagnose BrS? Can we explain the male predominance in the prevalence of BrS? Can we establish genotype-phenotype correlations for minor BrS-associated mutations? How can we further elucidate the pathophysiological mechanisms of BrS? Do age and sex play a predictive role in risk stratification of BrS? And, most importantly, how can we overcome this serious disease?

## 3. The Potential Role of Zebrafish in Research on Brugada Syndrome

In the present section, we will elucidate the potential role of zebrafish in BrS research by describing their general strengths and limitations as well as their cardiac electrophysiology. Also, how zebrafish have functioned as a model system for BrS in each of the studies published thus far will be discussed.

### 3.1 General Strengths and Limitations of the Zebrafish

The zebrafish (*Danio rerio*), a small tropical fish, has become a widely used animal model in the last three decades and has been used for various studies of human diseases, including several cardiac diseases [97, 98, 99] and, more specifically, studies of the electrophysiology of cardiac ion channel disorders [100, 101, 102]. The zebrafish is known to share several similarities with humans in terms of cardiac electrophysiology and has therefore become popular in the last decade for the study of cardiac arrhythmias. Compared to other experimental models that have been used to study BrS, the zebrafish has several advantages. However, like these other models, the zebrafish also has its limitations [29].

#### 3.1.1 General Strengths

Experimental models used in BrS research are mice, rabbits, dogs, and pigs, as well as heterologous expression systems, such as HEK cells and hiPSC-CMs [24, 26]. Although hiPSC-CMs show an emerging role in BrS research [25], the advantage of using an animal model is that the functioning and malfunctioning of a relevant gene can be studied in its physiological environment [24]. In addition, zebrafish have important advantages over other animal models. To start with, zebrafish have a relatively small size (3–5 cm) and are easy to breed. Their fecundity is high, as a single mating session can result in 200–300 embryos per week for each female. Thus, a major advantage is that breeding and maintenance of zebrafish are inexpensive and uncomplicated [103], especially in contrast to dog, pig, and rabbit models [26], making the zebrafish a cost-efficient animal model that allows for high-throughput screening [97, 101, 103]. In addition, the early embryo appears transparent, allowing direct observation of heart rate and rhythm [104]. Interestingly, genome-wide association analyses identified nine novel susceptibility variants for BrS near genes that play a major role in heart development and the control of cardiac ion channel expression [33]. The fact that cardiac development of zebrafish can be well tracked without complicated technological applications may be advantageous for further research on these variants.

Three days post-fertilization, all organs of the hatched animal will have matured, and 90 days post-fertilization, the fish will have developed into an adult animal [97]. This is particularly advantageous in contrast to porcine models, which have a long reproductive cycle [24, 26]. Furthermore, zebrafish have a substantially slower heart rate compared to the mouse, a commonly used model for studying BrS [26]. As the zebrafish heart beats 120–180 times per minute, compared to 300–600 beats per minute in mice, the heart rate of zebrafish is closer to that of humans (60–100 beats per minute) [97]. Moreover, the zebrafish genome has been completely mapped, and a comparison of the zebrafish reference genome with the human genome revealed that approximately 70% of human genes have one or more zebrafish orthologs [105]. Thereby, zebrafish provide a suitable opportunity for fast *in vivo *determination of the function of specific genes of interest, due to fast and effective gene knock-out techniques [101]. Concerning research of BrS, this is an appealing advantage, because a genotype-phenotype correlation could only be established for mutations in *SCN5A *thus far (see Section 2.2.1). More information on the potentially causative effects of mutations in BrS-associated genes could be collected using zebrafish as a knock-out model.

#### 3.1.2 General Limitations

Although the above advantages show great potential, the most prominent disadvantage of zebrafish is that it is not a mammalian species, which means that these fish are less related to humans than any of the aforementioned animal models. In addition, zebrafish are cold-blooded and live in an aquatic environment, indicating that their physiology is not identical to that of humans in many respects [104]. Also, compared to other vertebrates, zebrafish have undergone an additional cycle of whole genome duplication during evolution, resulting in a gene being present in two copies, which may make it more difficult to identify the functional role of a particular gene [105]. Moreover, the zebrafish heart has a two-chamber morphology, including a single atrium and a single ventricle, so that a distinction between the right and left sides of the heart cannot be made. This may limit any interpretation of data obtained from zebrafish regarding BrS, as BrS is a right ventricle-specific disease [24].

### 3.2 The Cardiac Electrophysiology of the Zebrafish

#### 3.2.1 Phenotyping Techniques for Zebrafish Cardiac Electrophysiology

Cardiac electrical activity, as discussed below in Sections 3.2.2 to 3.2.4, can be measured from intact zebrafish as well as from excised whole hearts, heart slices, and single cardiomyocytes using a variety of electrophysiological techniques, as recently reviewed in detail by Lin *et al*. [106] and Sieliwonczyk *et al*. [107]. In short, the tools for electrical phenotyping include *in vivo* and *ex vivo* ECG recording, optical mapping, sharp microelectrode techniques, and patch clamp methodology, all with their typical advantages and disadvantages. *In vivo* ECG recordings have the advantage of physiological neurohormonal regulation, normal drug absorption and metabolism, and intact peripheral circulation and respiration, while such conditions are typically absent in *ex vivo* ECG measurements, resulting in more controlled and standardized measurement conditions without the presence of anesthetic drugs [106, 108]. *In vivo* ECG recordings can be performed using ready-to-use commercially available systems, such as the ZS-200 Zebrafish system from iWorx Systems (Dover, NH, USA) or the ZF-01 CardioFish system from MDE GmbH (München, Germany), or by collection of custom laboratory and electrophysiological equipment and software [108, 109]. The latter approach has the advantage of also allowing *ex vivo* ECG recordings [106] or allowing sharp microelectrode measurements in excised zebrafish hearts, tissues or heart slices to record tissue-specific action potentials from atria or ventricles [106, 110], while cells remain in a normal relationship with neighboring cells [111]. For optical mapping experiments, preparations must contain contractile inhibitors, which pose several potential problems (for review, see Lin *et al*. [106]), to prevent movement [107], and must be loaded with voltage-sensitive dyes. Today, genetically encoded reporters are also available to determine voltage activity [112]. The aforementioned techniques are excellent electrophysiological phenotyping tools for whole hearts and multicellular preparations, in contrast to patch clamp recordings, which require single cells [113]. To this end, hearts or tissues are enzymatically dissociated and the resulting isolated cells can be measured directly or even cultured for long periods of time [114, 115]. The advantage of the patch clamp method is the controlled extracellular and intracellular solution composition, in combination with allowing detailed measurements and analysis of action potentials, membrane current densities, and biophysical properties, without the interference of other cells or neurohormonal regulation [113, 116]. In addition, single channel activity can be recorded [113] and drug experiments can be performed relatively easily without diffusion barriers.

#### 3.2.2 The Zebrafish ECG

**Fig. 3.**
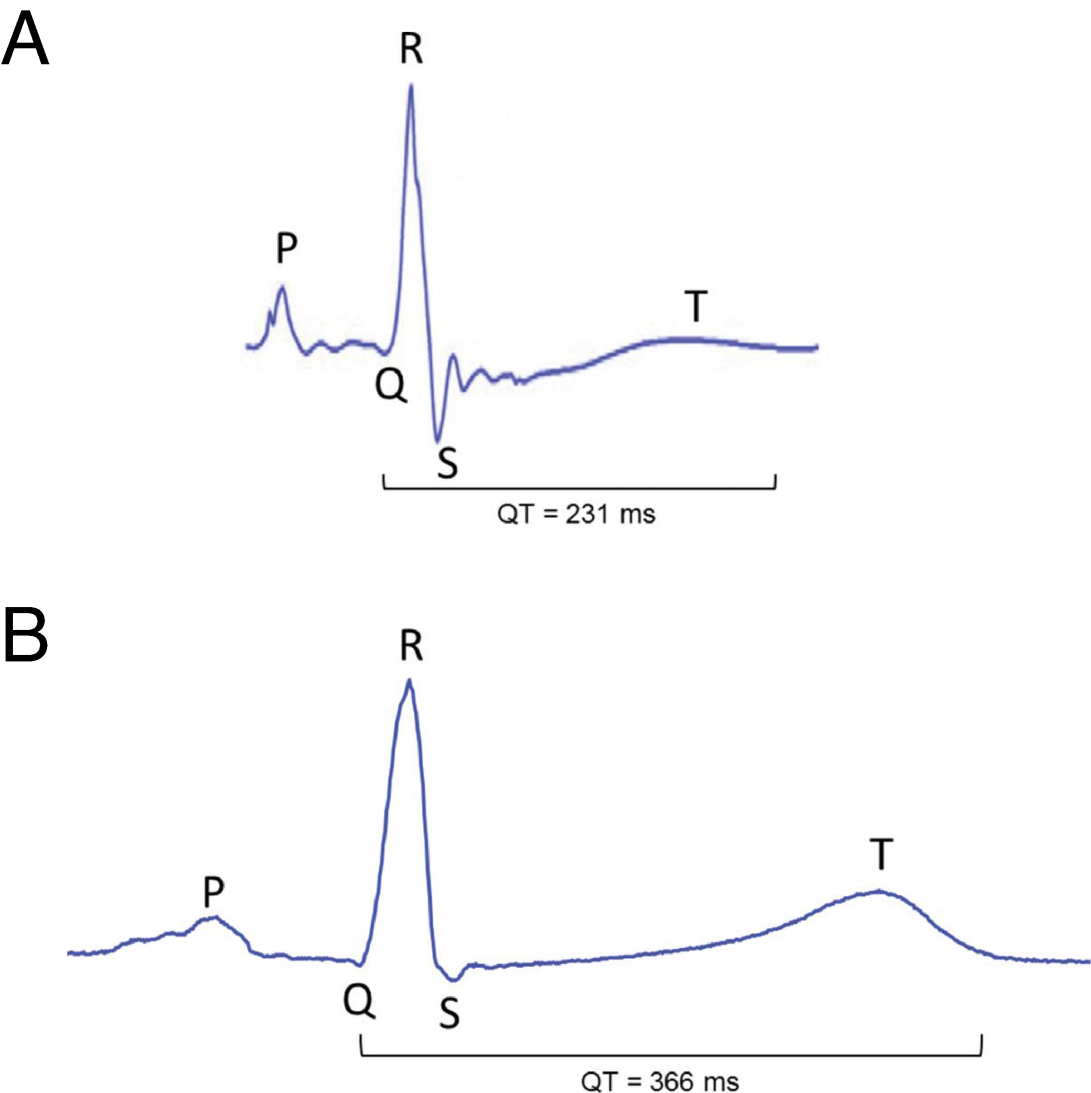
**Zebrafish and human electrocardiograms (ECGs) on an identical time scale**. (A) ECG of an adult zebrafish at 23 °C. (B) ECG of a healthy 43-year old human male. P and T indicate the P wave and the T wave, respectively, whereas Q, R, and S indicate the QRS complex, and QT indicates the QT interval. Reproduced with permission from Vornanen and Hassinen [29].

In 2006, Milan *et al*. [117] succeeded in recording the basal *in vivo* zebrafish ECG and subsequent research has contributed to further resolve the characteristics of the zebrafish ECG. Interesting similarities have been found between the zebrafish and human ECG, including a clearly visible P wave, QRS complex, and T wave [30], as illustrated in Fig. 3 (Ref. [29]). This suggests similarities in depolarization and repolarization between zebrafish and humans [101]. In addition, when hyperkalemia was induced in zebrafish, the changes in their ECG correlated with those observed in humans [109]. This may provide opportunities to identify new pharmacological drug treatments that act on the depolarization and/or repolarization of the cardiac action potential, as zebrafish may serve as a suitable whole-organism high-throughput screening model to assess the efficacy and safety of potential drugs for BrS [103].

#### 3.2.3 The Zebrafish Cardiac Action Potential

**Fig. 4.**
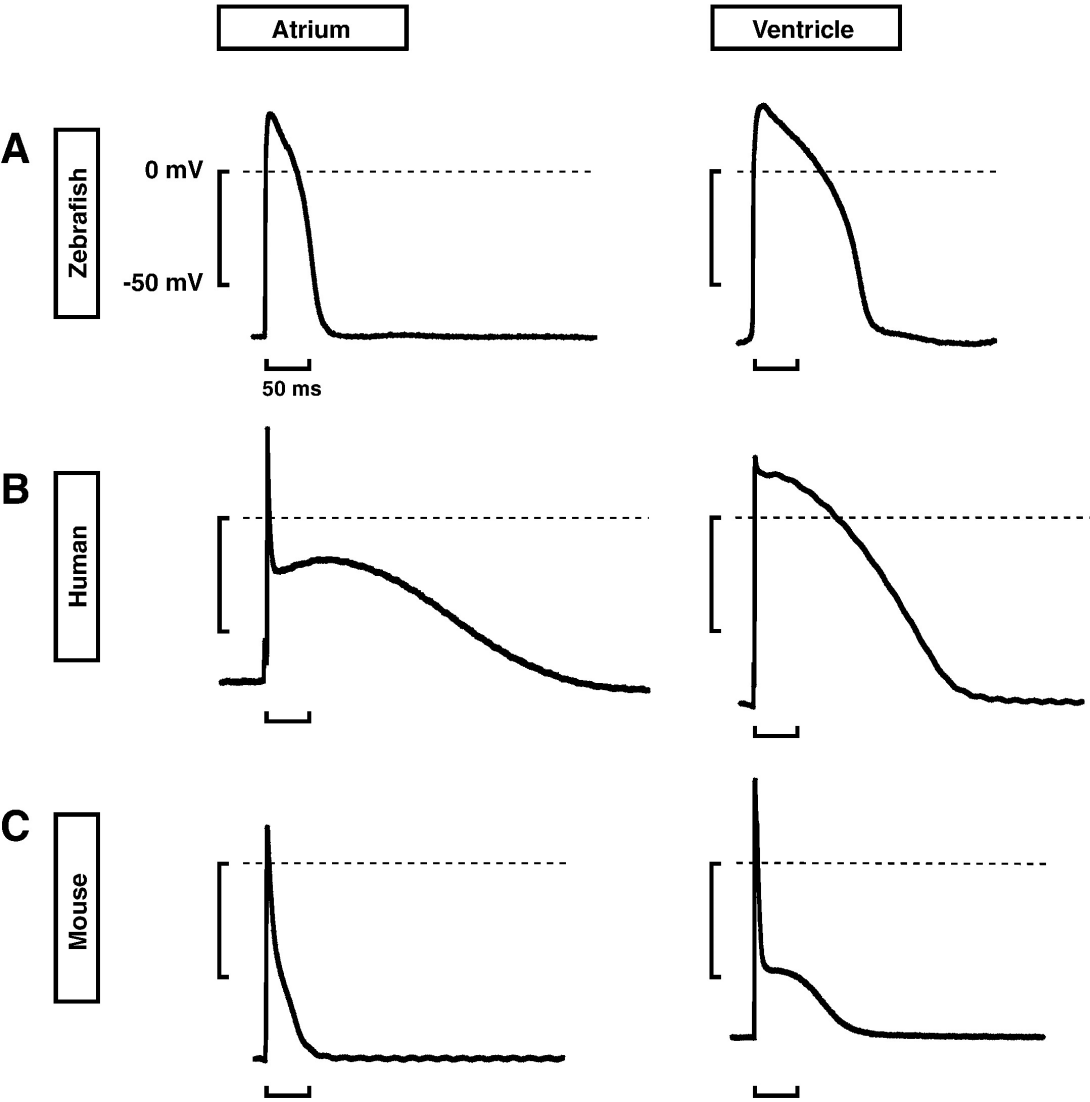
**Typical (A) zebrafish, (B) human, and (C) mouse atrial (left) and ventricular (right) action potentials on an identical time scale**. Zebrafish action potentials were recorded from intact hearts at the spontaneous beating frequency of ≈150 beats/min and at a physiological temperature of 28 °C, whereas the human right atrial and right ventricular and mouse left atrial and right ventricular action potentials were recorded from cardiac tissue stimulated at 1 Hz and at a physiological temperature of 37 °C. Reproduced with permission from Nemtsas *et al*. [110].

The zebrafish cardiac action potential shows striking similarities to that of humans. First, the resting membrane potential of zebrafish atrial and ventricular cardiomyocytes is not significantly different from that of human atrial and ventricular myocytes (Fig. 4A,B, Ref. [110]). Second, the shape of the zebrafish ventricular action potential (Fig. 4A, right) is highly similar to that of humans (Fig. 4B, right). It shows a fast upstroke depolarization phase, albeit with a substantially lower maximum upstroke velocity [110] (likely due to the lower recording temperature [118]), it contains a plateau phase, and it includes a rapid repolarization phase. In contrast, the spike-and-dome morphology observed in human action potentials (Fig. 4B) is lacking in zebrafish (Fig. 4A), due to the absence of a phase 1 early repolarization [29, 110]. Mice, on the other hand, have a much more pronounced ‘spike’ in their cardiac action potentials than humans, and they lack a distinct plateau phase (Fig. 4C). Taken together, the overall shape of the zebrafish cardiac action potentials (Fig. 4A) is more similar to that of humans (Fig. 4B) than that of mice (Fig. 4C) [30].

#### 3.2.4 Ion Current Profile of Adult Zebrafish Ventricular Myocytes

In zebrafish, the fast depolarization phase (phase 0) of the action potential is, as in humans, largely due to an inward I_Na_ [110, 118]. In adult zebrafish, the two main orthologous genes of *SCN5A *encoding the pore-forming α-subunit of the cardiac sodium channel are *scn5Lab* and *scn4ab*, which encode the Na_V_1.5Lb and Na_V_1.4b proteins, respectively [118]. With transcript levels of 65% in the atrium and 83% in the ventricle, Na_V_1.5Lb is the main transcript in both the atrium and ventricle. Na_V_1.4b shows transcript levels of values of 34% and 16%, respectively, whereas the total expression level of the remaining six transcripts is <1% [118]. Adult zebrafish ventricular cardiomyocytes also express the late sodium current (I_Na,late_) [119], as do human ventricular cardiomyocytes [120].

In contrast to humans, adult zebrafish atrial and ventricular cardiomyocytes also express a robust T-type calcium current (I_CaT_) [110, 121]. This I_CaT_ is carried by Ca_V_3.1 channels, encoded by the α*1G* gene, in both the atrium and ventricle [121]. With a peak density of ≈6.3 pA/pF at –30 mV (and at 28 °C) in ventricular myocytes [121], its contribution to the upstroke of the ventricular action potential is small, as long as I_Na_ is not strongly reduced. I_CaL_ is mainly carried by Ca_V_1.2 channels, encoded by the α*1C* gene, in both the atrium and the ventricle [121]. In ventricular myocytes, it has a peak density of ≈7.7 pA/pF at 0 mV (and at 28 °C), similar to that of I_CaT_. In human ventricular myocytes, the peak density of I_CaL_ is ≈10 pA/pF (at 37 °C) [122], which is of a similar magnitude, also considering the difference in recording temperature.

Cardiac delayed rectifier potassium channels have not yet been studied in the same detail as the sodium and calcium channels. Langheinrich *et al*. [123] demonstrated that zebrafish embryos express the *zerg* ortholog of *HERG* (also known as *KCNH2*, encoding the pore-forming HERG α-subunit of the I_Kr_ channel) and are sensitive to QT-prolonging drugs, suggesting that I_Kr_ is expressed at a functional level in the embryonic zebrafish ventricle. In subsequent studies on embryonic and adult zebrafish ventricular cardiomyocytes, I_Kr_ was found to be the main repolarizing current, whereas a functional I_Ks_ could not be detected [110, 124]. In a more recent study, however, transcripts of the *kcnq1* and *kcne1* genes, as orthologs of *KCNQ1* and *KCNE1*, were detected in the zebrafish atrial and ventricular myocardium and an associated functional I_Ks_ was detected in adult zebrafish ventricular cardiomyocytes [125]. Both atrial and ventricular zebrafish cardiomyocytes express an inward rectifier potassium current (I_K1_) with its typical electrophysiological characteristics [126]. In the human heart, the main Kir2 subunit is Kir2.1 in both the atria and ventricles, with some expression of Kir2.2 in the ventricles and Kir2.3 in the atria [127]. In the zebrafish, however, the main cardiac Kir2 subunits are drKir2.2a and drKir2.4, with transcript levels of 65% and 29%, respectively, in the atrium and 6% and 93%, respectively, in the ventricle [126].

In line with the absence of a spike-and-dome action potential morphology, Nemtsas *et al*. [110] did not detect a functional I_to_ in their adult zebrafish ventricular myocytes, whereas Alday *et al*. [124] did not observe a functional effect of the specific I_to_ channel blocker heteropodatoxin in their study on embryonic zebrafish ventricular myocytes. Alday *et al*. [124] confirmed the absence of K_V_4.3 channel proteins, which constitute the pore of the I_to_ channels in human ventricular cardiomyocytes, in the cell membrane by immunofluorescence staining. Thus, a functional I_to_ could not be established in zebrafish ventricular cardiomyocytes, despite the presence of the *KCND3* (human potassium voltage-gated channel subfamily D member 3 gene, encoding K_V_4.3 in human) ortholog *kcnd3* in the zebrafish genome, suggesting its presence and function in tissues other than the heart. This lack of ventricular I_to_ may be a limitation in studying the pathophysiological mechanism of BrS, as the BrS repolarization hypothesis states that a reduced I_Na_ leads to a local premature repolarization (‘loss of dome’) due to a dominant I_to_. Actually, two gain-of-function mutations in* KCND3*, rather than loss-of-function mutations in* SCN5A*, were associated with BrS in a study by Giudicessi *et al*. [128].

### 3.3 Published Findings on BrS from Zebrafish Models

Over the years, a considerable number of reviews have been published on the cardiac electrophysiology of zebrafish and on the potential of zebrafish for the study of cardiac arrhythmias (*e.g.*, [27, 28, 29, 30, 97, 98, 99, 100, 101, 102, 103, 129, 130, 131, 132, 133, 134]). However, despite this anticipated potential, the actual use of zebrafish in such studies is limited, as shown by Sieliwonczyk *et al*. [107], who carried out a comprehensive PubMed search in February 2021 for original papers (*i.e.*, not reviews) on studies using zebrafish to study ventricular arrhythmias. They ended up with a total of only 32 papers. The most frequently studied disorder appeared to be the long QT syndrome, with a total of nine papers.

In June 2023, we used the PubMed and Google Scholar databases to search for ‘Brugada’ and ‘zebrafish’ in combination and select all papers in which zebrafish were actually used as model system for BrS thus far. This search returned only the papers by Zhou *et al*. [31], Juang *et al*. [32], and Barc *et al*. [33], from 2016, 2020, and 2022, respectively. We repeated our search in January 2024 to check for any recently added papers and found only one additional paper, which is the paper by Chiang* et al*. [34] that was published in January 2024. The number of four papers using zebrafish as a model for BrS seems limited, but we must keep in mind that the total number of original studies using zebrafish and ventricular arrhythmias is not high at all [107], which immediately underscores that zebrafish are still an emerging animal model for cardiac arrhythmias, including studies of BrS. One reason may be that zebrafish, like most animal models, may not present all pathophysiological conditions of a human cardiac syndrome [101, 132]. While keeping this in mind, we will now discuss how zebrafish have already contributed to new insights into BrS in these four studies in order to provide a better and more specific understanding of how these animals can actually be used to investigate aspects of BrS.

#### 3.3.1 Obtaining Evidence for a Pathophysiological Role of a Mutation in MOG1 (Mitochondrial Outer Membrane Protein 1) Associated with BrS

In 2008, Wu *et al*. [135] revealed that the MOG1 protein, encoded by the *MOG1* gene, is not only involved in the trafficking of macromolecules into and out of the cell nucleus, but also in the cell surface expression of Na_V_1.5. In 2011, Kattygnarath *et al*. [136] presented experimental data supporting the hypothesis that dominant-negative mutations in *MOG1* can impair the trafficking of Na_V_1.5 to the cell membrane and thereby reduce I_Na_, which would define *MOG1* as a potential susceptibility gene for BrS. Building on these studies, Zhou *et al*. [31] further investigated the physiological role of MOG1 using zebrafish as an *in vivo *model. In one of their experiments, they aimed to test whether the BrS-associated E83D mutation in the *MOG1* gene would affect the heart rate of zebrafish embryos. One-cell stage zebrafish embryos were injected with mRNA samples transcribed from a wild-type human *MOG1 *expression plasmid, whereas two-cell stage embryos were injected with mRNA samples transcribed from a mutant human *MOG1 *expression plasmid containing the E83D mutation. At a dose of 400 pg mRNA, they observed a significantly reduced heart rate in zebrafish embryos overexpressing the E83D mutant MOG1, compared to the embryos overexpressing the wild-type MOG1 and compared to the embryos injected with control *EGFP *mRNA. These data thus provide further evidence that the potentially BrS-associated E83D mutation in *MOG1* is a functional mutation that affects MOG1 function. Although this E83D mutation in *MOG1*has only been identified in a single BrS patient, and a causative role has not yet been established, zebrafish made an important contribution to this study by functioning as a suitable *in vivo *model. In the future, zebrafish may further contribute to research aimed at elucidating the causative role of *MOG1 *mutations in BrS. Besides, zebrafish may be suitable for investigating MOG1, delivered by gene therapy, as an effective treatment option for cardiac arrhythmias with an impaired I_Na_, including BrS [31].

#### 3.3.2 Characterization of Functions of CNVs (Copy Number Variants) Associated with BrS

CNVs are repeated sequences in the genome that have different numbers of repeats among individuals within a species. They provide diversity within a species, but some CNVs are known to contribute to genetic disorders [137]. As the role of CNVs in susceptibility to BrS was unknown, Juang *et al*. [32] aimed to identify a CNV that is associated with BrS. In their study, 335 unrelated Taiwanese BrS patients were enrolled. They observed a copy number deletion of *GSTM3 * (human glutathione S-transferase mu 3 gene) in 23.9% of these BrS patients, while this deletion was found in only 0.8% of 15,829 controls in the Taiwan BioBank. Among the BrS patients, carriers of this *GSTM3 *deletion showed higher risks of sudden cardiac arrest and syncope than non-carriers. The function of this deletion was determined using HEK cells and zebrafish. Experiments in HEK cells revealed that the *GSTM3 *deletion probably causes a reduced antioxidant effect, resulting in a reduced I_Na_. More pertinent, *gstm3 *knockout male adult zebrafish were created using the CRISPR/Cas9 technique and the susceptibility to induced VT or VF was assessed in wild-type,* gstm3^+⁣/-^*, and *gstm3^-⁣/-^* zebrafish, using 21 zebrafish from each group. No VT/VF could be induced in zebrafish from the wild-type group, both at baseline and in the presence of 0.1–10 µM of the sodium channel blocker flecainide that was used as a provocative drug. However, four of the *gstm3^+⁣/-^* and six of the *gstm3^-⁣/-^* zebrafish already exhibited inducible VT/VF at baseline and these numbers increased with increasing concentrations of flecainide, up to eight and ten, respectively, in the *gstm3^+⁣/-^* and *gstm3^-⁣/-^* groups. Thus, the number of knockout zebrafish with inducible ventricular arrhythmias was significantly higher in the *gstm3^+⁣/-^* and *gstm3^-⁣/-^* groups than in the wild-type group, both in the absence and in the presence of flecainide. Interestingly, Juang *et al*. [32] also administered quinidine to their zebrafish and observed that the number of *gstm3^+⁣/-^* and *gstm3^-⁣/-^* zebrafish with inducible VT/VF reduced upon quinidine infusion, in line with the clinical effects of quinidine in BrS patients [86] mentioned in Section 2.3.2.

The findings of Juang *et al*. [32] suggest that the deletion of *GSTM3 *may affect the risk of arrhythmias in BrS patients, and that testing for this mutation might contribute to improved risk stratification for BrS patients [32]. In their study, the zebrafish had a great added value by functioning as a model system for further assessment of the *in vivo* function of *GSTM3* as a CNV associated with BrS.

#### 3.3.3 Revealing MAPRE2 (Human Microtubule Associated Protein RP/EB Family Member 2 Gene) as a Potential Gene Involved in the Pathophysiology of BrS

In their genome-wide association analyses, Barc *et al*. [33] had identified novel risk loci for BrS, including *MAPRE2*, which is a gene encoding a protein that regulates the microtubule organization. However, the function of microtubule organization in ion channel trafficking had not yet been investigated. Therefore, they generated *mapre2 *knockout zebrafish using CRISPR/Cas9 and isolated the hearts from 5-day postfertilization *mapre2 *knockout and wild-type larvae. Using optical mapping, they observed a significantly lower conduction velocity and action potential upstroke velocity in the *mapre2 *knockout hearts compared to the control. Together with a significantly lower upstroke velocity observed in *MAPRE2 *knockout hiPSC-CMs, this strongly suggested that *MAPRE2 *knockout leads to a reduced I_Na_, which they confirmed in patch clamp measurements in hiPSC-CMs. Together with a previous study showing that axonal targeting of the human Kv1 ion channel is dependent on a microtubule plus-end binding protein [138], these findings led Barc *et al*. [33] to suggest that modulation of microtubule function may be involved in the molecular pathophysiology of BrS, as this may affect Na_V_1.5 channel trafficking. In brief, in the study by Barc *et al.* [33], zebrafish contributed to highlighting a novel mechanism underlying BrS susceptibility, by functioning as a model system to investigate the effects of dysfunction of the protein encoded by *MAPRE2*.

Interestingly, *mapre1 *knockout results in similar I_Na_ and action potential upstroke velocity changes in zebrafish and hiPSC-CMs as reported for *mapre2 *knockout [139], but so far no associations with BrS have been made. The electrophysiological defects of *mapre1 *knockout could be rescued by the drug SB216763, which is a glycogen synthase kinase 3β inhibitor, and preliminary results have suggested that it also rescues ECG abnormalities in zebrafish with *mapre2* dysfunction [140].

#### 3.3.4 Further Research into the Function of MAPRE2

Recently, the work of Barc *et al*. [33] on* MAPRE2* was extended by the study of Chiang* et al*. [34]. Using high-resolution optical voltage mapping on zebrafish hearts isolated from both germline knockout and morpholino-injected lines at 5 days post-fertilization, they confirmed that *mapre2 *loss of function phenocopies Na_V_ loss of function in larval hearts. They recorded ECGs from anesthetized adult wild-type and *mapre2* knockout zebrafish and found a significant increase in QRS duration in the heterozygous knockout fish and even more so in the homozygotes, without apparent changes in other ECG parameters. Voltage clamp experiments in single ventricular myocytes isolated from adult zebrafish revealed that I_Na_ did not show changes in its kinetics, but that its density was significantly reduced in the *mapre2* knockout myocytes, in line with the increase in QRS duration. Remarkably, however, RT-PCR (Reverse Transcription Polymerase Chain Reaction) experiments showed no significant difference in the mRNA level of all major ion channel genes, including the* scn12aa *and *scn12ab* homologs of *SCN5A*. In line with this, Western blotting did not reveal a significant difference in expression of the main cardiac Na_V_ channel encoded by *scn12ab*.

Experiments on the gross ventricular structure and contractility of embryonic and adult zebrafish showed that these were not different between *mapre2* knockout and wild-type zebrafish. However, immunostaining demonstrated that *mapre2* loss of function leads to disruption of adherens junctions, which is likely to affect Na_V_ channel function, given their ‘anchoring’ in the intercalated disc [141]. Further mechanistic experiments learned that *mapre2* loss of function results in changes in microtubule dynamics. In brief, the zebrafish played a prominent role in the multidimensional study by Chiang* et al*. [34], which elucidated how loss-of-function mutations in *MAPRE2* may result in a reduced I_Na_, identifying such mutations as a genetic risk factor for BrS.

## 4. Conclusions

In our review, the strengths and limitations of the zebrafish as a model system for BrS have been described. Transgenic expression of human genes in zebrafish has been shown to be successful, and researchers have been able to generate gene knockout zebrafish using CRISPR/Cas9. Using these strategies, zebrafish could contribute to explaining novel genetic factors in the susceptibility to BrS, and perhaps explain BrS as an oligogenic disease [52]. In addition, the humanization of zebrafish provides opportunities for screening potential pharmacological compounds. By replacing a gene with its human ortholog, important information about drug-target interactions can be obtained, providing insights into the efficacy and safety of potential drug treatments [30].

Screening potential BrS drugs with the use of zebrafish is a challenge for the future. In this future research, it should not be overlooked that “sex and gender matters to the heart” [142]. In this regard, the deliberate use of male zebrafish is a remarkable aspect of the study by Juang *et al*. [32]. Although this may be understandable because the prevalence of BrS is higher in males, the female sex should not be completely excluded from future research on BrS. It is important to prevent “knowledge gaps” [142] as much as possible by including the female sex in BrS research. However, as long as certain studies remain proof-of-principle, this issue should not cause major complications for translational medicine.

In the context of translational medicine, it is also important to emphasize the general limitation of using animal models to study human diseases. Due to the interspecies variability between humans and animals, it is not possible to fully translate findings from animal research to human physiology [30]. Due to the higher homology between humans and mice, compared to humans and zebrafish, zebrafish cannot completely replace the murine animal model [97]. However, zebrafish are able to reduce the number of mammals required for experiments by serving as a suitable additional model system. Thus, the strongest argument for using zebrafish as a model system for BrS is the fact that zebrafish can be very valuable in combination with other model systems. In particular, the combination with hiPSC-CMs shows potential, because this will provide evidence based on human material and the zebrafish will provide evidence as a highly suitable *in vivo *system.

Despite its limited kinship with humans, its two-chamber heart morphology, and its lack of I_to_, four extensive studies [31, 32, 33, 34] have already shown that zebrafish can function as a valuable model system in research on BrS by applying gene knockout strategies, and by letting zebrafish embryos express a human protein with a BrS-associated mutation. Hence, without doubt, future studies will contribute to highlighting the potential of zebrafish for research on BrS, ultimately leading to new insights into this serious arrhythmogenic disease.
